# Feasibility of continuous non-invasive delivery of oxygen monitoring in cardiac surgical patients: a proof-of-concept preliminary study

**DOI:** 10.1186/s12871-024-02561-2

**Published:** 2024-05-25

**Authors:** Roderica R. G. Ng, Suneel R. Desai, Felicia S. W. Chu, Ming Ann Sim, Sheryl W. L. Chee, Jerry Y. H. Fuh, Lian-Kah Ti, Sophia T. H. Chew

**Affiliations:** 1 Department of Anaesthesiology, Singapore General Hospital, Singapore, Singapore; 2 Duke-NUS Medical School, Singapore, Singapore; 3 Departments of Surgical Intensive Care and Anaesthesiology, Singapore General Hospital, Singapore, Singapore; 4 Department of Anaesthesia, National University Health System, Singapore, Singapore; 5 Department of Mechanical Engineering, National University of Singapore, Singapore, Singapore; 6 Yong Loo Lin School of Medicine, National University of Singapore, Singapore, Singapore

**Keywords:** Biomedical technology, Cardiac surgery, Hemodynamic monitoring, Oxygen delivery, Perioperative outcomes

## Abstract

**Purpose:**

Oxygen delivery (DO_2_) and its monitoring are highlighted to aid postoperative goal directed therapy (GDT) to improve perioperative outcomes such as acute kidney injury (AKI) after high-risk cardiac surgeries associated with multiple morbidities and mortality. However, DO_2_ monitoring is neither routine nor done postoperatively, and current methods are invasive and only produce intermittent DO_2_ trends. Hence, we proposed a novel algorithm that simultaneously integrates cardiac output (CO), hemoglobin (Hb) and oxygen saturation (SpO_2_) from the Edwards Life Sciences ClearSight System® and Masimo SET Pulse CO-Oximetry® to produce a continuous, real-time DO_2_ trend.

**Methods:**

Our algorithm was built systematically with 4 components – machine interface to draw data with PuTTY, data extraction with parsing, data synchronization, and real-time DO_2_ presentation using a graphic-user interface. Hb readings were validated.

**Results:**

Our algorithm was implemented successfully in 93% (*n* = 57 out of 61) of our recruited cardiac surgical patients. DO_2_ trends and AKI were studied.

**Conclusion:**

We demonstrated a novel proof-of-concept and feasibility of continuous, real-time, non-invasive DO_2_ monitoring, with each patient serving as their own control. Our study also lays the foundation for future investigations aimed at identifying personalized critical DO_2_ thresholds and optimizing DO_2_ as an integral part of GDT to enhance outcomes in perioperative cardiac surgery.

**Supplementary Information:**

The online version contains supplementary material available at 10.1186/s12871-024-02561-2.

## Introduction

Worldwide, about 2 million open heart surgeries are performed annually [1] and these high-risk cardiac surgeries are associated with multiple complications and increased morbidity and mortality [2]. Optimizing oxygen delivery (DO_2_) has been shown to improve perioperative outcomes for cardiac surgery [3–6].

DO_2_ optimization during cardiopulmonary bypass (CPB), as part of goal-directed therapy (GDT), has demonstrated its efficacy in mitigating stage 1 acute kidney injury (AKI) following cardiac surgery [4]. Additionally, the use of functional hemodynamic monitoring within the Kidney Disease: Improving Global Outcomes (KDIGO) bundle during the initial 48 h post-cardiac surgery has shown a significant reduction of moderate and severe AKI [7]. However, continuous DO_2_ monitoring is not routine, and it does not extend into the postoperative period.

DO_2_ can be calculated using the oxygen flux equation, which is represented by the simplified formula: ${DO}_{2}= CO\times Hb \times {SpO}_{2} \times 1.34$, where CO is the cardiac output, Hb is the hemoglobin concentration, SpO_2_ is the oxygen saturation, and 1.34 is the maximal binding oxygen carrying capacity of Hb.

Traditionally, the pulmonary artery catheter (PAC) is the gold standard to measure CO based on thermodilution techniques. However, the insertion of the PAC is invasive, and various studies have reported that PAC use increases the likelihood of arrhythmia, pulmonary artery rupture, thrombosis, and sepsis [8–10]. Technical inaccuracies in PAC monitoring can also occur due to catheter misplacement, injectate temperature, thermistor malfunction, and clotting at the catheter tip [11]. To compute DO_2_, blood sampling of the hemoglobin is done, and DO_2_ is then derived from the oxygen flux equation as mentioned above. However, blood sampling of Hb is intermittent, and the monitoring of DO_2_ is non-continuous.

With the advent of new technology, the feasibility of non-invasive, continuous CO and Hb monitoring are now possible. The ClearSight System® (ECS, Edwards Life Sciences) measures blood pressure non-invasively and determines CO via continuous pressure waveform analysis [12]. Similarly, the Masimo SET Pulse CO-Oximetry® (MSPC) technology has enabled the continuous, non-invasive monitoring of Hb and oxygen saturation (SpO_2_) which mitigates the problems of intermittent Hb sampling [13]. Both new systems have improved greatly in portability and ease of operation.

As both ECS and MSPC operate on separate systems, current users will have to manually multiply components of the oxygen flux equation (namely CO, Hb, and SpO_2_) to derive DO_2_, hence making it cumbersome and impossible to have real-time DO_2_ readings. Therefore, we propose an algorithm that simultaneously integrates CO readings from the ECS with SpHb and SpO_2_ readings from the MSPC to produce real-time DO_2_ trends and evaluate the feasibility of continuous non-invasive delivery of oxygen monitoring in cardiac surgical patients in a proof-of-concept study.

Our proposed algorithm holds significant potential to revolutionize perioperative management in cardiac surgery by providing clinicians with the ability to engage in personalized medicine, where real-time insights into a patient’s DO_2_ trend will enable tailored interventions based on individual patient needs. This personalized approach to monitoring and optimization has the potential to mitigate complications such as AKI and improve overall patient outcomes.

## Materials and methods

### Components to build the real-time DO_2_ monitoring system

**Fig. 1:**
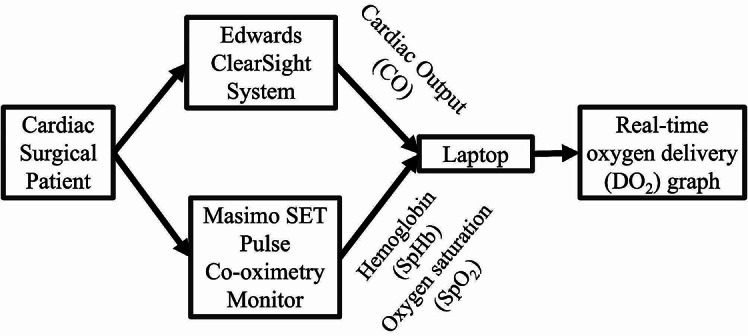
Physical setup of devices to a laptop for processing and to obtain a real-time DO_2_ trend graph

To build our real-time, non-invasive DO_2_ monitoring system, 4 components were built systematically from the machine interface, to data extraction, to data synchronization and integration, and then the real-time data presentation using the Graphical User Interface (GUI). Figure 1 illustrates the step-by-step flow of information from a cardiac surgical patient, acquired through the ECS and MSPC, to the laptop for processing using our novel algorithm, ultimately generating the real-time DO_2_ trend graph using GUI.

### Machine interface

The ECS and MSPC are from different companies, and they operate on their proprietary operating systems. Therefore, an interface was required for data extraction. We devised the use of a free open-source terminal emulator and serial console application called PuTTY to draw data from these two machines by connecting them via serial ports to a main computer. This allows the output from both ECS and MSPC to be directly drawn to the computer for further processing by the algorithm (Fig. 1).

### Data extraction

Each output line from the MSPC has individual timestamps with labels corresponding to the various parameters. This allows for easy parsing of data as the SpO_2_ and SpHb values are easily identified and isolated. However, output lines from the ECS did not have timestamps, proper indentations, and/or data labeling. Therefore, parsing was then applied based on the positioning of the CO data value that corresponded to the CO value displayed on the machine and the CO data could be isolated from the output lines.

### Data synchronization and integration

In light of the differing time intervals between the outputs of both monitors, with the MSPC operating twice as fast as the ECS (i.e. MSPC every 1 s and ECS every 2 s), direct utilization of the extracted output to derive accurate DO_2_ readings was unfeasible. Therefore, in the final data extraction algorithm, MSPC outputs were aligned with ECS outputs every 2 s to synchronize the derivation of DO_2_. Data integration between the ECS and MSPC was done using Python codes to obtain real-time DO_2_ which was calculated using the oxygen flux equation, as delineated in the Introduction.

### Real-time data presentation (GUI)

**Fig. 2:**
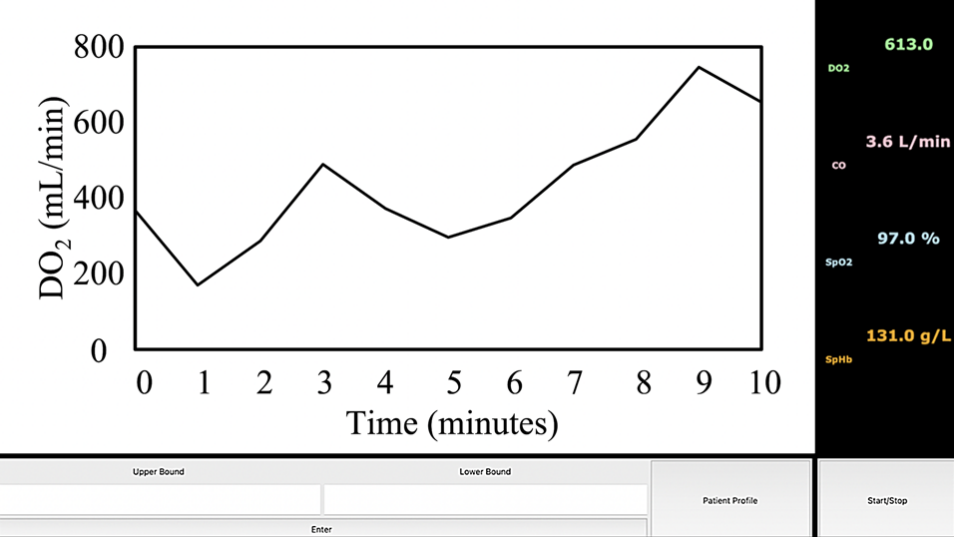
Graphical user interface (GUI) displaying a real-time DO_2_ trend

Real-time DO_2_ values are graphed as a dynamic trend chart that updates every 20 s, offering users an easy-to-follow overview of the data, enabling them to swiftly detect any anomalies and respond promptly. The real-time GUI was built using tkinter, which is event-based and calls for functions when individual widgets are triggered by the user. Widgets included in the GUI include frame, window, canvas, top level, button, label and check button. Prior to testing the algorithm on cardiac surgery patients, a simulator was also created by randomizing the different parameters (CO, SpHb and SpO_2_) within their normal ranges to obtain a real-time DO_2_ graph trend shown in Fig. 2.

### Patient eligibility criteria

The inclusion criteria included patients aged 21 years and above undergoing elective cardiac surgery in a tertiary cardiac center. The exclusion criteria included emergency cases, patients on postoperative intra-aortic balloon pumps, pregnant women, and those who declined participation.

### Clinical care and application of ECS and MSPC monitors

Routine perioperative clinical care was provided to all recruited patients as per institutional protocol. The attending cardiac anesthesiologist would exercise discretion in determining whether to adjust patient treatment based on real-time derived DO_2_ values perioperatively.

All patients had an ECS finger cuff placed on the index finger and an MSPC adhesive sensor placed on the ring finger before surgery to obtain baseline DO_2_ values and to ensure no equipment malfunction. The ECS and MSPC monitors, along with our laptop integrating the signals, remained powered on, and running continuously from the baseline measurement until the patient was extubated, or up to a maximum of 8 h postoperatively, except during CPB and patient transfer from the operating theatre (OT) to the intensive care unit (ICU).

There was no explicit recommendation for data integration from the manufacturers of each device.

### Collection and analysis of primary outcome

Baseline patient characteristics, intraoperative variables, and anesthetic charts were obtained from routine clinical records. These clinical data were analyzed descriptively using numbers (percentages) for categorical variables and mean (standard deviation) or median (interquartile range) for continuous variables, depending on data distribution.

We also obtained the raw data from the ECS and MSPC monitoring devices, as well as the derived DO_2_ data based on the algorithm, from the main computer. The application PuTTY was used to draw data from both ECS and MSPC, and an in-built algorithm was used to derive DO_2_. The percentage of successful implementation of our algorithm was calculated.

### Collection and analysis of exploratory secondary outcome and safety

Considering that DO_2_ is a modifiable risk factor for AKI, a common complication post-cardiac surgery [3, 4], we trended DO_2_ values for all patients, tabulated the perioperative DO_2_ parameters using descriptive statistics, and studied the graphical representation of DO_2_ trends between patients who developed AKI following cardiac surgery and those without. AKI was defined by the KDIGO criteria. An unpaired 2-sample T test was done to ascertain if differences in perioperative DO_2_ parameters between patients with and without postoperative AKI reached statistical significance (*p* < 0.05). Safety data on the use of ECS finger cuffs and MSPC adhesive sensors were also collected.

### Validation of SpHb readings

SpHb readings from the MSPC were validated against laboratory Hb using a paired 2-sample T-test, where a statistically non-significant result (*p* > 0.05) suggests the validity of the SpHb readings. The agreement between the MSPC SpHb and laboratory Hb readings was further corroborated by the Bland-Altman analysis. The mean and differences were calculated for each matched pair of MSPC SpHb and laboratory Hb readings. Subsequently, the bias and limits of agreement were determined. A Bland-Altman plot, illustrating the difference versus the mean of SpHb and laboratory Hb values, was generated to visually assess the distribution and patterns of agreement. A 1-sample T-test was conducted to assess the statistical significance of the bias, where a statistically non-significant result (*p* > 0.05) suggests agreement between the 2 readings.

All data analyses were done using IBM SPSS Statistics (version 26.0, Armonk NY).

## Results

### Patient recruitment, baseline patient characteristics and intraoperative variables

61 patients met the eligibility criteria of the study and were recruited between 3 August 2021 and 5 October 2021. Baseline patient characteristics and intraoperative variables are summarized in Table 1.

**Table 1:** Baseline patient characteristics and intraoperative variables of eligible patients

	Value
**Preoperative variables**	
Age (years)	63.1 ± 8.7
Gender: Male	52 (85.3%)
Body mass index (kg/m^2^)	25.1 (22.5–27.2)
Ethnicity	
Chinese	39 (63.9%)
Malay	12 (19.7%)
Indian	8 (13.1%)
Others	2 (3.3%)
ASA status	
ASA 2	1 (1.6%)
ASA 3	56 (91.8%)
ASA 4	3 (4.9%)
ASA 5	1 (1.6%)
Hypertension	35 (57.4%)
Diabetes	31 (50.8%)
History of cardiac arrhythmia	4 (6.6%)
History of cerebrovascular events	3 (4.9%)
Left ventricular ejection fraction (%)	53.3 ± 9.8
EuroSCORE II	0.9 (0.7–1.6)
Preoperative creatinine (mg/dL)	1.0 (0.8–1.1)
Preoperative estimated glomerular filtration rate (ml/min/1.73m^2^)	86.4 ± 19.3
**Intraoperative variables**	
Type of cardiac surgery	
Coronary artery bypass graft surgery	56 (91.8%)
Coronary artery bypass graft and valve surgery	5 (8.2%)
Cardiopulmonary bypass time (min)	83.4 (68.5–108.0)
Aortic cross clamp time (min)	49.5 (41.0–110.0)

Continuous variables are presented as mean ± standard deviation or median (25th − 75th percentile) if they are normally distributed or skewed respectively. Categorical variables are presented as numbers (percentages)

The American Society of Anesthesiologists (ASA) physical status classification system categorizes patients based on their overall health and comorbidities in the pre-anesthetic setting, while the EuroSCORE II scoring system predicts the risk of in-patient mortality for patients undergoing cardiac surgery

### Implementation of algorithm for cardiac surgical patients

Our novel algorithm was successfully implemented in 57 cardiac surgical patients, generating complete DO_2_ trends with individual continuous DO_2_ trend graphs for each patient. The mean perioperative DO_2_ values obtained across all individuals in this study ranged from 219.4 to 704.7 mL/min/m^2^, with an average monitoring duration of 378 ± 93 min. The unsuccessful implementation of the algorithm on 4 patients was due to undetectable CO signals due to machine interface problems and cuff malfunctions.

### DO_2_ and AKI

Based on the KDIGO criteria for AKI, the incidence of postoperative AKI was 18.0% (*n* = 11). The perioperative DO_2_ parameters between patients with and without postoperative AKI are summarized in Table 2. The averages of mean DO_2_ and 10th centile DO_2_ were lower in patients with postoperative AKI compared to those without, though this did not reach statistical significance (*p* > 0.05).

**Table 2:** Perioperative DO_2_ parameters between patients with and without postoperative AKI

Perioperative DO_2_ parameters	Overall (*n* = 57)	AKI (*n* = 11)	No AKI (*n* = 46)	*p* value
Average of mean DO_2_ (mL/min/m^2^)	371.1 ± 91.2	346.6 ± 83.6	376.9 ± 92.9	0.326
Average of 10th centile DO_2_ (mL/min/m^2^)	280.2 ± 81.5	248.7 ± 80.2	287.8 ± 80.8	0.154
Average monitoring duration (min)	378.1 ± 92.8	410.9 ± 104.4	369.0 ± 88.7	-

Continuous variables are presented as mean ± standard deviation as they are normally distributed

The averages of the 10th centile DO_2_ among the study population and its subgroups provide an important aggregated measure of the lower boundary of DO_2_ distribution, as oxygen delivery, which is critical to prevent organ dysfunction, may be impaired at low DO_2_ levels

**Fig. 3:**
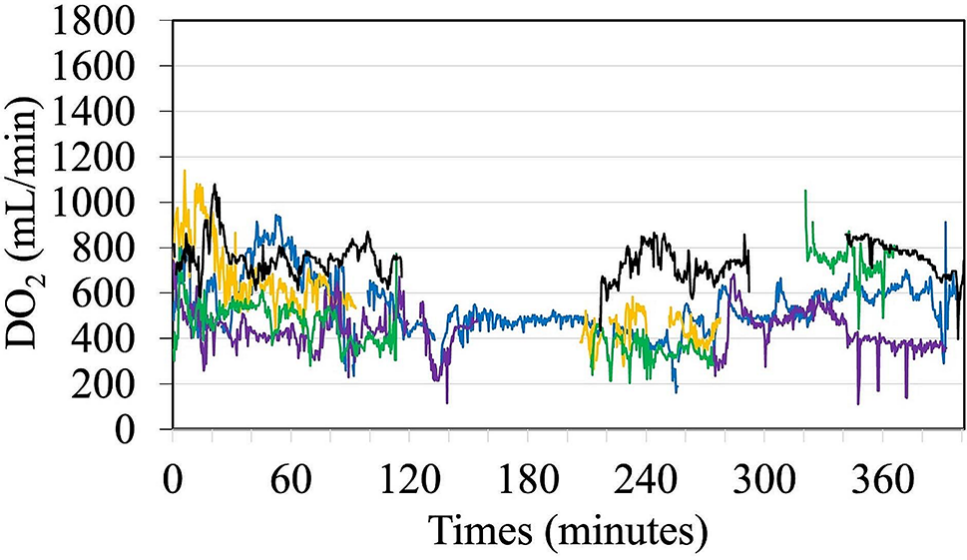
DO_2_ trend graph with time in patients with postoperative AKI (each represented with a different color), where downtrends of postoperative DO_2_ and/or multiple DO_2_ dips are seen

Visually, the DO_2_ trends in patients with postoperative AKI are shown in Fig. 3, where downtrends of postoperative DO_2_ and/or multiple DO_2_ dips are noted. A case example of a patient with postoperative AKI, where the DO_2_ trend was largely influenced by low SpHb values, is presented in Supplementary Fig. 1.

**Fig. 4:**
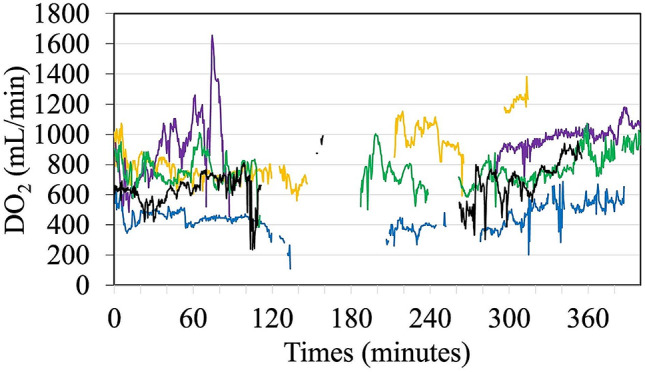
DO_2_ trend graph with time in patients with no postoperative AKI (each represented with a different color), where uptrends of postoperative DO_2_ are seen

In comparison, Fig. 4 shows the uptrending of postoperative DO_2_ trends in patients without postoperative AKI. A case example of a patient without postoperative AKI, where the DO_*2*_ trend was jointly influenced by CO and SpHb values, is presented in Supplementary Fig. 2.

### Safety outcomes

Two patients violated protocol by wearing the ECS sensor for an average of 645 min, surpassing the recommended limit of 480 min. Subsequently, these patients developed cyanotic fingers, which resolved spontaneously when cuff measurements were discontinued. Additionally, one of these patients had a skin abrasion, which was successfully managed with conservative management.

### Validation of SpHb readings

A paired 2-sample T-test between laboratory Hb values and corresponding MSPC Hb readings found no significant difference (10.6±1.4 g/dL vs. 10.5±1.6 g/dL respectively, *p* = 0.770).

This result was further corroborated with the Bland-Altman analysis. The bias, representing the average difference of MSPC SpHb and laboratory Hb readings, was − 0.05 ± 1.29 g/dL, with upper and lower limits of agreement at 2.46 g/dL and − 2.57 g/dL, respectively (Supplementary Fig. 3). The one-sample T-test comparing the mean difference to 0 yielded a non-significant p-value of 0.771, indicating agreement between the 2 measurement methods.

## Discussion

This is the first proof-of-concept study to show the feasibility of continuous, non-invasive delivery of oxygen monitoring in cardiac surgical patients with our novel algorithm that simultaneously integrates CO readings from the ECS with SpHb and SpO_2_ readings from the MSPC to produce continuous, real-time DO_2_ readings non-invasively. Moreover, each patient’s preoperative baseline DO_2_ value served as their reference point, providing ease for the real-time interpretation of oxygen delivery and allowing for monitoring of the DO_2_ trend.

### Utility of DO_2_ monitoring

The ultimate goal of hemodynamic monitoring and management is to provide adequate DO_2_ to meet the metabolic oxygen demands of the tissues and organs, to avoid anaerobic metabolism and accumulation of lactic acidosis. However, there are limitations to the use of DO_2_ monitoring in current clinical practice and functional hemodynamic monitoring is largely used as a surrogate for DO_2_ instead.

### Addressing current limitations

Our novel algorithm can have real-time DO_2_ monitoring and seeks to address the current limitations faced. For non-surgical and non-cardiac surgical patients, DO_2_ is not routinely measured as CO is commonly obtained via minimally invasive (e.g. pulse pressure analysis method) or invasive means (e.g. pulmonary artery catheter bolus thermodilution method), and Hb sampling is done only intermittently via point-of-care or laboratory tests. The utility of DO_2_ is limited as it is cumbersome due to the need for invasive lines and the lack of continuous DO_2_ readings and trends.

Successful integration of CO, Hb and SpO_2_ readings to obtain DO_2_ readings at 1- and 10-minute intervals using the Quantum Perfusion System and Dideco software system respectively, during cardiac surgery has been previously reported [5, 14]. However, this can only be done when the patient is on CPB when CO is dependent on the pump flow setting. Maintaining DO_2_ above 272 mL/min/m^2^ during CPB has been shown to improve postoperative outcomes [15] but DO_2_ trending cannot be extended to the post-CPB period due to technical limitations. Instead, post-CPB monitoring relied largely on functional hemodynamic monitoring which cannot track end-organ DO_2_. Our solution of a non-invasive, continuous DO_2_ monitoring system is the first to demonstrate that it can be used to track DO_2_ postoperatively and to demonstrate its use in predicting poor outcomes after cardiac surgery.

Our setup is easy to use, non-invasive, and continuous, allowing real-time trending of DO_2_. This ensures that the physician is alerted early to a possible downturn in patients’ parameters, and this DO_2_ trending can be expanded to a large array of at-risk patients across different disciplines and clinical care settings.

### Robustness of novel algorithm

While recognizing concerns about expanding the use of monitors beyond their original purpose, the robustness of our novel algorithm is built upon the established reliability of the proprietary algorithms embedded in the ECS and MSPC systems.

The non-invasive CO monitor by ECS has been evaluated and validated to be equivalent to its invasive counterpart during and after cardiac surgery – the pulmonary artery catheter bolus thermodilution method [16]. Simultaneously, given the unparalleled ability to have continuous and non-invasive SpHb monitoring unlike any other available hemoglobin monitors, the SpHb monitor by MSPC has been highly recommended as a trend monitor and allows for more effective blood utilization in cardiovascular surgery [17]. Additionally, we corroborated the accuracy of hemoglobin measurements obtained by MSPC through a comparison with laboratory test results.

However, it is important to note that calibration of the ECS and MSPC systems pre- and postoperatively should be carried out by the manufacturer’s recommendations to prevent system drift and inaccurate readings.

### DO_2_ and AKI

We have presented both statistical and graphical comparisons of DO_2_ trends between cardiac surgical patients with and without postoperative AKI. As anticipated, patients with postoperative AKI exhibited poorer DO_2_ values overall. These results lead to the important question regarding the critical DO_2_ threshold in the post-CPB period which would reduce the incidence of AKI. Having demonstrated the feasibility of DO_2_ trending, our next focus would be to demonstrate and validate its robustness as a monitor for detecting AKI-associated DO_2_ trends and/or critical threshold. This endeavor aims to enhance the optimization of DO_2_ to mitigate and reduce postoperative adverse outcomes.

The PrevAKI randomized controlled trial has previously shown that using functional hemodynamic monitoring with early GDT optimization can significantly reduce postoperative moderate to severe AKI [7]. Our proposed solution of DO_2_ trending not only looks at the functional hemodynamic parameters but also addresses the adequacy of oxygenation and blood management which will enhance GDT optimization.

### Advancing patient-centric care: personalized critical DO_2_ threshold

Critical DO_2_ values of < 280 ml/min/m^2^ during CPB have consistently been shown to be associated with AKI after cardiac surgery [4]. Currently, there is a paucity of studies on the optimal postoperative critical DO_2_ threshold, which reduces the risk of postoperative complications. Published critical DO_2_ values so far are based on the study cohort baseline characteristics, the outcome measures, and the statistical modeling method used [5, 14].

To address this gap, our innovative setup empowers clinicians with unprecedented access to continuous, real-time DO_2_ monitoring. This technological leap facilitates the identification of a patient’s unique baseline DO_2_ and the analysis of DO_2_ trends in relation to postoperative outcomes. This capability unlocks the potential for personalized critical DO_2_ thresholds, marking a paradigm shift towards a patient-centric model of care. The real-time availability of personalized DO_2_ readings serves as an unparalleled reference point, enabling clinicians to monitor trends and detect potential complications early. The personalized baseline not only enhances precision in identifying subtle changes in oxygen delivery tailored to each individual but also allows for interventions based on the patient’s distinct physiological response. As we continue to pursue technological refinements, the prospect of trending DO_2_ off-site and implementing customized alarms for each patient becomes increasingly significant. This not only ensures timely interventions but also empowers healthcare providers to proactively address deviations from a patient’s personalized baseline using early GDT strategies, ultimately improving postoperative adverse outcomes.

### Limitations

There are a few limitations to our study. Our study was conducted within a single centre and involved a relatively small number of cardiac surgical patients. To enhance the external validity of our observations and extend the generalizability of our conclusions beyond the confines of cardiac surgical care, future studies should strive for broader inclusivity by encompassing non-cardiac surgical patients. Expanding the scope to include a more diverse patient population will contribute to a more comprehensive understanding of the implications of continuous DO_2_ monitoring across various clinical contexts.

Additionally, continuous, real-time DO_2_ monitoring introduces specific challenges. Firstly, 2 separate proprietary monitors (ECS and MSPC) are currently required to integrate and obtain DO_2_ readings, though each monitor is not bulky. Secondly, the ECS finger cuffs and MSPC adhesive sensors are costly, however, the cost may potentially be reduced if these consumables are purchased in bulk when DO_2_ monitoring is routinely done. Thirdly, the maximum duration of continuous DO_2_ readings is limited to 8 h at one stretch according to manufacturers’ recommendations. Lastly, the use of these peripheral monitors is less useful in very low perfusion states, e.g. during CPB, but continuous DO_2_ readings can be obtained from the perfusion pump machine.

### Future works and applications

Our novel setup and algorithm show great promise in revolutionizing patient monitoring systems to include non-invasive DO_2_ trend monitoring as the gold standard for hemodynamic optimization. Further refinement to our algorithm to allow the inclusion of user inputs of a patient’s body surface area will provide real-time indexed DO_2_ values to allow for better referencing, standardization, and comparability between patients due to variations in body surface area. Also, incorporating an intelligent alert system will provide timely audio and visual cues to draw the attention of the clinician when DO_2_ values are below a critical threshold. In addition, future studies should validate non-invasive ECS-derived CO measurements against established methods like bolus thermodilution or echocardiography, extending the utility of our novel algorithm to include the use of individual DO_2_ values.

The vast amount of continuous DO_2_ data and its components will allow us to build a robust predictive model of postoperative outcomes utilizing machine learning and artificial intelligence. This will allow for a tailored response, such as optimizing Hb and/or hemodynamic parameters, for individual patients at specific timepoints, significantly improving postoperative outcomes.

Apart from the intensive care setting, this non-invasive DO_2_ trending can be easily performed in all clinical care areas for at-risk patients as an early trend monitor to guide decisions on when to escalate care and aid in appropriate utilization of scarce resources – all of which value adds the clinical care being delivered.

## Conclusions

We have demonstrated the feasibility of the use of the first non-invasive, continuous DO_2_ trend monitor which simultaneously integrates CO readings and SpHb readings from two separate monitors, with each person serving as their own control, in a proof-of-concept study.

We also presented lower overall DO_2_ trends in patients with AKI following cardiac surgery. In the future, identification of personalized critical DO_2_ thresholds and the optimization of DO_2_ as part of GDT therapy will be explored to improve postoperative outcomes after cardiac surgery.

### Electronic supplementary material

Below is the link to the electronic supplementary material.

Supplementary Material 1

## Data Availability

The code utilized for extracting and integrating data from the ECS and MSPC is available on Github, Inc (https://doi.org/10.5281/zenodo.7936133). The code is open-sourced and can be freely accessed and reused for non-commercial and commercial purposes under the GNU General Public License, with permission from the corresponding author.

## Declarations

### Study registration

The study was registered on 20 May 2021 at clinicaltrials.gov (NCT04895384).
