# Supplementary material for: Feasibility of continuous non-invasive delivery of oxygen monitoring in cardiac surgical patients: a proof-of-concept preliminary study
Source: BMC Anesthesiol. 2024 May 25;24:187. doi: 10.1186/s12871-024-02561-2 (PMC11127411; doi:10.1186/s12871-024-02561-2)
Supplement: Supplementary file 1 — Supplementary Material 1 [file 12871_2024_2561_MOESM1_ESM.docx]

**Supplementary Figures**


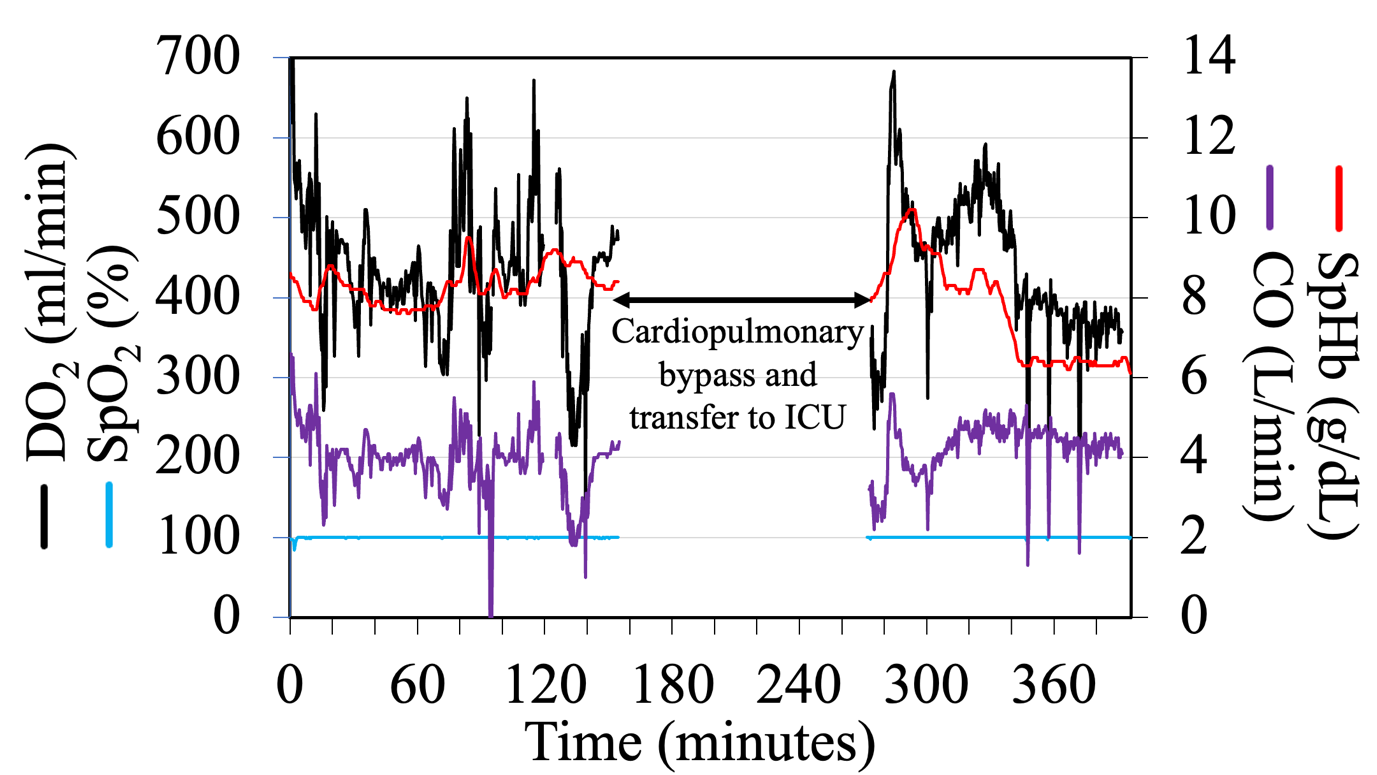


**Supplementary Figure 1.** A case example of a patient with postoperative AKI, where the DO_2_ trend was largely influenced by low SpHb values, is presented in Supplementary Figure 1. Downtrending of postoperative DO_2_ is seen with multiple dips.

The left y-axis is used for DO_2_ and SpO_2_, while the right Y-axis is used for SpHb and CO.


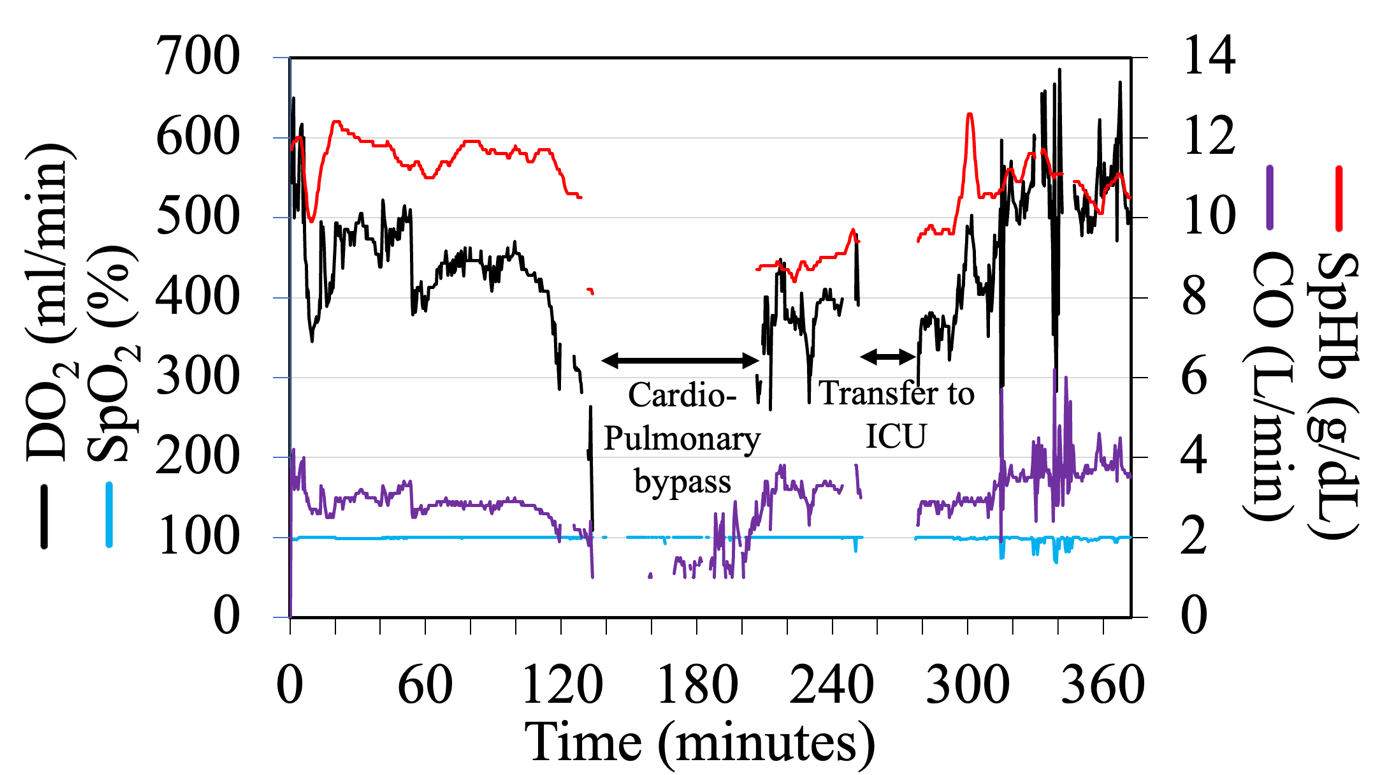


**Supplementary Figure 2.** A case example of a patient without postoperative AKI, where the DO_2_ trend was jointly influenced by CO and SpHb values, is presented in Supplementary Figure 2. Uptrending of postoperative DO_2_ is seen.

The left y-axis is used for DO_2_ and SpO_2_, while the right Y-axis is used for SpHb and CO.


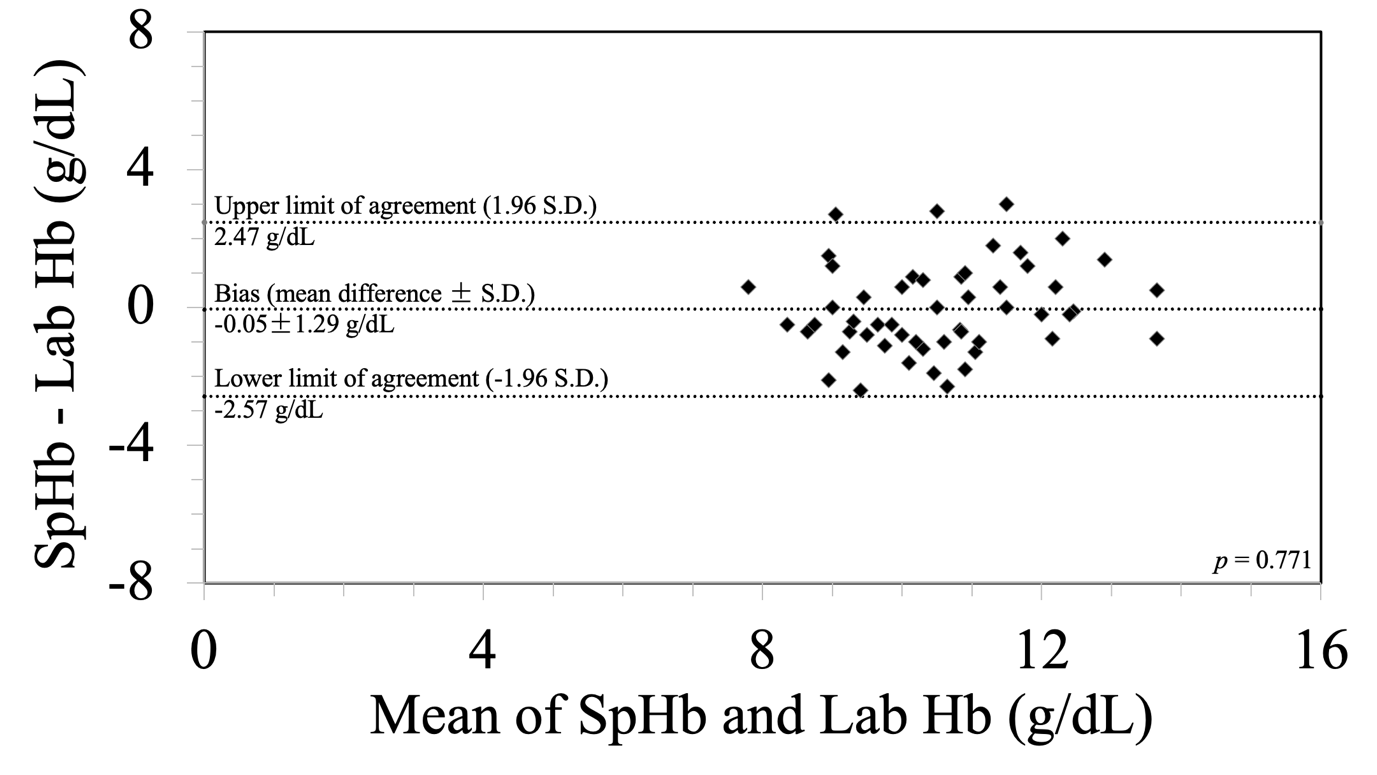


**Supplementary Figure 3.** Bland-Altman plot for MSPC SpHb and laboratory Hb readings scattered between the upper and lower limits of agreement. The 1-sample T test comparing the mean difference of the readings to 0 yielded a non-significant p-value of 0.771, indicating agreement between the 2 measurement methods.
